# The Carey Coombs Memorial Lecture on the Pathology and Surgical Treatment of Cardiac Ischæmia

**Published:** 1937

**Authors:** Laurence O'Shaughnessy

**Affiliations:** Consultant Surgeon to the Lambeth Cardiovascular Clinic (L.C.C.), London


### The Bristol Medico-Chirurgical Journal

"*Scire est nescire, nisi id me
Scire alius sciret.*"

SUMMER, 1937.

### THE CAREY COOMBS MEMORIAL LECTURE

DELIVERED AT A MEETING OF THE SOCIETY
ON WEDNESDAY, MAY 5th, 1937.

THE PRESIDENT (Dr. R. C. CLARKE)
*in the Chair.*

BY

LAURENCE O'SHAUGHNESSY, F.R.C.S.,
*Consultant Surgeon to the Lambeth Cardiovascular Clinic (L.C.C.), London.*

ON

### THE PATHOLOGY AND SURGICAL TREATMENT OF CARDIAC ISCHÆMIA.

In the first place I should like to express my gratitude for the opportunity of delivering this lecture. It is quite clearly a lecture which could better be delivered by many more competent authorities than myself; for it is a lecture in memory of a great physician who spent his active life in the study of cardiovascular disease, while I have merely laboured on the fringes of the subject, and that only of recent years.

I was already familiar with some of Coombs's writings, and it has been a most welcome task during the last few weeks to review in closer detail those which I had not previously read. It soon became apparent to me that many of the more significant aspects of his work could not be dealt with in this lecture. There was his discovery of the "submiliary nodule" in rheumatic carditis, his important contributions to the pathology and treatment of rheumatism, and his valuable work on its social and economic importance. There was also his very beautiful and detailed description of some of the rarer manifestations of cardiovascular disease — I refer especially to his important paper on abnormalities of the pulmonary artery. However, on reading his Lumleian Lecture for 1930 I was struck by the accuracy with which the pathology of cardiac ischæmia was described, and in some of his other papers I have looked for further evidence of his views on coronary disease, a subject which is relevant to our work in the Lambeth Cardiovascular Clinic. I mean to show very briefly how his views have been confirmed and amplified by subsequent clinical and experimental work.

Anginal pain is common to all types of cardiac ischæmia, and in 1930 the "aortic" theory of cardiac pain, of which Sir Clifford Allbutt had been so able an exponent, had still its strong adherents. Coombs however asserted that anginal pain originated in the heart itself, for he had observed that the anginal pain of syphilitic aortitis differed in no way from the angina associated with generalized atheroma of the coronary tree ; and while it might well be that the aortic plexus could be involved in aortitis, this was clearly not the case in coronary sclerosis. The one factor common to all types of coronary insufficiency was a defective supply of oxygen to the myocardial cells, and Coombs therefore considered that interference with cell metabolism caused cardiac pain. There is much recent work to support this view—the clinical researches of Sir Thomas Lewis may be cited.

In syphilitic coronary insufficiency there is a normal peripheral coronary tree—a very beautiful injection specimen prepared by Dr. Bruce Perry illustrates this particular point in the lecture; but as Coombs himself expressed it, the coronary blood supply is strangled at its source, for the orifices of the vessels are obstructed, and in addition loss of the normal elasticity of the wall of the aorta, together with deformity in varying degree of the aortic valves, disturbs the normal mechanism by which blood is fed to the coronary tree. In coronary atheroma Coombs believed that it was loss of the elasticity of the coronary vessels which rendered them incapable of those subtle variations which adequate function of this vital segment of the vascular tree demands. Loss of elasticity in its most extreme form is, of course, seen in calcification of the coronary vessels. It was from this type of cardiac ischæmia that John Hunter died. Coombs had seen coronary thrombosis occur as a complication of coronary atheroma, and he appreciated to the full the part which some incidental infection, especially of the respiratory tract, may play in determining the moment at which an actual deposit of clot on some roughened area of the vessel wall may cause complete obstruction of its lumen.

The anginal pain of severe anæmia Coombs explained on the grounds that here, although distribution of the blood supply to the heart could proceed normally, the fluid supplied was incapable of providing the myocardium with that rich oxygen supply which under all conditions normal cardiac action requires.

The most striking evidence of the importance of cardiac ischæmia as a common factor in a variety of heart affections has been provided by Büchner, Weber and Haager. Their monograph contains a careful record of forty-three fatal cases of cardiac ischæmia and correlation of the clinical findings, including, of course, electrocardiographic studies, with the post-mortem appearances both macroscopic and microscopic. Although in many of their cases anginal pain was a prominent symptom this was not invariable, and in some cases dyspnœa on attempted exertion, recurrent attacks of pulmonary œdema or attacks of congestive heart failure provided the evidence for impaired cardiac function. The problem of the ischæmic heart, which may progress even to the point of perforation of an infarct without pain, is one of the most fascinating in cardio-vascular pathology, and it may be that its solution lies in more systematic examination of the autonomic nervous system in such cases. Their series includes obvious cases of coronary thrombosis in an otherwise normal coronary tree, thrombosis complicating atheroma of the coronary arteries, cases of hypertensive heart failure with gross atheroma of the tree but no actual obstruction, fatal cases of syphilitic aortitis when only the orifices of the coronary vessels were affected, and even one or two cases of heart failure following chronic bronchitis and emphysema in the presence of coronary atheroma. In all this group of cases there were characteristic electrocardiograms, and at autopsy careful examination of the heart revealed pathological changes in the myocardium. After obstruction of a main coronary artery gross evidence of an old or recent infarct was invariably present, and in other cases, where no actual block of the circuit was present but merely a general impairment of its lumen, scattered areas of recent necrosis or old fibrosis could invariably be detected by serial sections.

As Coombs pointed out, there is one condition in which angina may be a prominent symptom despite normal coronary arteries, and this is severe anæmia. Büchner in his ingenious experiments provided a rational explanation of this apparent inconsistency. He produced an intense anæmia in his rabbits by hæmorrhage and then set them to work on a treadmill. His animals died, and at autopsy multiple areas of necrosis were found scattered throughout the myocardium—a familiar finding in coronary obstruction in man. He obtained similar results if he reduced the oxygen-carrying capacity of the blood by inducing a state of carbon-monoxide poisoning, and thus the obvious fact that anoxæmia of the cardiac muscle is the determining cause of death in cardiac ischæmia received experimental confirmation.

Under some conditions coronary occlusion is a fatal event, but the organism is often in a position to effect a remarkable degree of natural compensation, so that even successive attacks of coronary thrombosis may be survived and sometimes an astonishing degree of normal activity may be regained in the interval, sometimes years in extent, between the initial and the second attack. Natural compensation is especially favoured, as Sir Clifford Allbutt long ago pointed out, if the process of coronary obliteration is one of slow onset; but even in Chiari's famous case of embolus of the right coronary artery (one of the few *clear* records in the literature of coronary embolism as distinct from thrombosis) his patient survived this insult for two days, when a second embolism of the left coronary artery abruptly terminated his life.

Natural compensation for occlusion of the coronary tree may be effected in several ways. Although it seems quite possible that there may be subtle compensatory mechanisms of which we know nothing, the mechanism of at least some of them is clear, and the following possibilities for the provision of an alternative blood supply exist :—

1. Anastomoses between the right and left coronary arteries may come into action when one or other main trunk is occluded.

2. The Thebesian vessels may act as an additional source of cardiac nutrition.

3. The natural collateral channels which connect the coronary tree with the vasa vasorum of the great vessels may become more pronounced.

4. The heart may acquire a new and additional blood supply if it adheres to the parietal pericardium.

1. *Inter-coronary Anastomoses.*

The free communications between the branches of the right and left coronary arteries, which were first effectively demonstrated in the exquisite preparations of Spalteholz, probably constitute the most important compensatory mechanism in cardiac ischæmia. That mechanism is effective in diffuse obstruction of the tree (athero-sclerosis and arterio-sclerosis), and it must play an essential part when actual occlusion of one of the main trunks (right coronary, descending branch of the left coronary, or circumflex) occurs. At the same time occlusion of a main trunk, whether it is sudden or gradual, is followed by an infarct of the heart wall. An explanation of the apparent inconsistency between the anatomical researches of Spalteholz and the effect of coronary occlusion as seen in the experimental animal or the living patient is provided by the ingenious experiments of Crainicianu. Crainicianu perfused the coronary trees of human hearts shortly after death under carefully-controlled conditions of pressure and temperature. He was able to show that the total volume of fluid perfused in a given time could be modified by experimental ligature of various branches of the tree, and also that the volume flow in a given time was less in hearts that were the seat of coronary disease. As he very reasonably points out, this method of examination will reveal what may be termed "physiological" obstruction in the capillary bed which histological examination may fail to demonstrate.

The degree of natural compensation by this means in an acute case of coronary occlusion probably depends, as in cases of peripheral vascular occlusion, on the restoration of an optimum blood-pressure, relaxation of any spasm in those portions of the coronary circuit still patent, and localization rather than extension of the thrombus; for an extending thrombosis will occlude side branches of the affected vessel and militate against the formation of a collateral circulation.

2. *The Thebesian Vessels.*

The Thebesian vessels are small channels which connect all four chambers of the heart with the venous and capillary bed of the coronary tree. Their morphology has been demonstrated in injection studies by Grant and Viko and by Wearn. The exact part played by these vessels in compensation for cardiac ischæmia is unknown, but there is some recent work which suggests that it may be an important one. Slater and Kornblum record a remarkable case of mitral stenosis complicated by bilateral coronary occlusion, in which good compensation was maintained at least for a time, and they suggest that the altered pressure relationship within the heart owing to the mitral lesion may have made the Thebesian circulation abnormally efficient. Leary and Wearn report two cases of syphilitic aortitis with bilateral coronary occlusion which seemed to have had good compensation, and suggest that in these patients the Thebesian vessels must have been of service. Nevertheless, these patients died of heart failure, and there is no pathological evidence to suggest that the Thebesian vessels can in any way replace the normal coronary circulation, although the possibility remains that they may assist in compensation for a defective circulation.

3. *The Natural Collateral Channels of the Coronary Tree.*

Minute arteries which connect the coronary arterial tree with the vasa vasorum of the aorta and the pulmonary artery were first demonstrated by Langer. He also found that material injected into the coronary tree could be recognized in the vessels of the mediastinum and the diaphragm, and even lying far out in the lung under the bronchial mucosa. An occasional and very interesting finding was the presence of a minute artery taking origin in the aorta some distance above the sinus of Valsalva and pursuing an independent course to ramify on the basal region of the heart. Langer's findings have been confirmed by more recent work, but few observations appear in the literature as to the state of these vessels in persons dead of cardiac ischæmia. Von Redwitz, however, noted in some of his exhaustive post-mortems the presence of a network of small vessels at the root of the aorta and pulmonary artery.

4. *The Formation of New Extra-Cardial Anastomoses.*

The heart can only acquire a new collateral blood supply if partial or complete destruction of the epicardium takes place, so that an adhesion may form between the heart and the parietal pericardium. This important mechanism is only available in those rare cases when the victim of coronary occlusion has previously suffered obliteration of his pericardial space as a result of an old pericarditis, or when following a coronary occlusion an actual infarct is produced with its base on the epicardial surface of the heart.

Examples of this extreme degree of natural compensation are very rare, but since Sternberg first recorded the syndrome of pericarditis epistenocardica there have been other reports of angina complicated by pericarditis with a favourable immediate outcome. Sternberg's patient was a man of 48 who suffered a coronary thrombosis, and in whom a clinical diagnosis of pericarditis was made by auscultation. He made a good recovery, and returned to active life for two years before dying of heart failure. At autopsy a large cardiac aneurysm was found adherent to the parietal pericardium. Mönckeberg cites other similar instances. One of these, a case published by Fujinami, is very striking. Sir Clifford Allbutt recorded the case of a doctor who suffered a severe attack of angina pectoris, after which he was bedridden for some time, and during his illness pericardial friction was detected. He recovered and returned to active practice. The opposite picture was presented by two cases of cardiac aneurysm of which I had personal experience. So far as can be determined the site of vascular obstruction was the same as in the cases cited, but in neither case had pericardial adhesions formed. One patient died from progressive heart failure and the other from hæmo-pericardium following perforation of the aneurysm into the pericardial sac a few months after the initial attack.

It is true that this type of compensation is available only to a small number of cases of coronary thrombosis. White only found it present eight times in 62 cases, and in Parkinson's and Bedford's 83 autopsies on coronary occlusion there were 11 cases of pericarditis, and in 100 cases examined clinically pericardial friction was only heard in 7. On the other hand, in Coombs's series of 148 cases of coronary thrombosis he detected pericardial friction on forty-two occasions; and although it was his opinion that the immediate mortality was higher in these cases, it would appear from his figures that the subsequent course of those patients who did survive was rather more favourable than in the other group. Of course, his period of observation had been short, and in view of the importance which this question has now assumed it would be of extreme interest to know the course run by his group of patients during the last five years.

Despite their comparative rarity, there is every indication of the practical importance of these adhesions when they are present. Wearn and his associates carried out injection studies on the human cadaver, and could demonstrate a vascular connection between the coronary tree and the parietal pericardium when adhesions were present. I was concerned in a case in which pericardectomy was carried out for *concretio cordis*. The patient died some three months later, and in the interval, although the mechanical relief afforded by the operation was demonstrated in a reduction of her ascites and venous pressure, an unusual type of cardiac irregularity had developed. After death histological examination showed scattered areas of necrosis and fibrosis in part of the auricular wall, from which the adherent pericardium had been stripped.

It is interesting to speculate on the relation of these natural reparative processes to the prognosis of the various types of cardiac ischæmia.

An established case of syphilitic aortitis should theoretically be of the greatest disadvantage, for here intercoronary anastomoses can be of little service if the orifices of both vessels are occluded, endarteritis of the vasa vasorum of the aorta and pulmonary artery is likely to render them less efficient as collateral channels, the heart remains free in the pericardium, and therefore additional collaterals cannot reach it, and so the only mode of compensation remaining is a rather doubtful Thebesian supply.

The gradual development of diffuse atheroma of the coronary tree should enable compensation to be established through the natural collateral channels, but unless a frank coronary thrombosis occurs late in the disease an infarct cannot form, and there is no chance of the natural acquisition of a new blood supply.

### THE TECHNIQUE OF CARDIO-OMENTOPEXY.

The following description illustrates one of the methods we have worked out at the Buckston Browne Farm Laboratory of the Royal College of Surgeons of England and in the Lambeth Cardio-vascular Clinic, by which the processes of natural compensation may be initiated and supplemented by operation.

After suitable premedication general anæsthesia is induced and maintained with the Tiegel-Henle apparatus, which supplies oxygen under positive pressure together with vaporized ether. With the patient on his back, the chest is entered through an incision along the fifth intercostal space extending from the midline to the anterior axillary line. The fifth and sixth costal cartilages are divided near the sternum after the manner of Kirschner and by the use of a large Sauerbruch intercostal retractor the pericardium is exposed. The phrenic nerve is identified and crushed with a hæmostat, and once this has been done the pressure in the anæsthetic apparatus is reduced (from 10 to 6 cm. of water) and the table is tilted to the right. As a result of these manœuvres the left leaf of the diaphragm appears in the operation field, and after the insertion of two sutures the muscle is incised. The abdomen is then explored through the diaphragmatic incision; a suitable portion of the omentum is obtained and brought through into the chest. The wound in the diaphragm is then closed by suture. The table is brought back to its original position, the degree of inflation of the lung is again increased, and the heart, covered by the pericardium, is once more in view. With caution the pericardium is incised and the graft is attached to the surface of the heart and to the edges of the pericardium by suture with fine linen thread. Finally the chest wound is closed in layers in the usual way.

### CASE HISTORY.

A missionary's widow, aged 65, had led a strenuous life for many years in high altitudes. Angina pectoris for twelve years: attacks had become more frequent and more severe, and she had been almost confined to bed for over eighteen months, being finally unable even to wash herself. (A sister had angina.) She was referred to the clinic by Dr. L. Lyne, of Hove, and was admitted on 1st October. Examination: very obese; heart enlarged to anterior axillary line; soft aortic systolic murmur and very short diastolic whiff; radiogram confirmed cardiac enlargement; B.P. 247/117; no œdema; electrocardiogram showed complete bundle-branch block with large amplitude of Q R S complexes in leads I and III.

After ten days' treatment with general massage, starch free diet, and administration of theamine and amytal, cardio-omentopexy was performed at Lambeth Hospital on 16th October, 1936, with the assistance of Dr. Mansell and Dr. Berry under general anæsthesia by Dr. Hasler. Aleuronat was injected into the pericardial sac and at the site of graft. At the end of operation the pulse-rate was 69, the B.P. 170/90, and the electrocardiogram showed no change. Recovery was interrupted by a paroxysm of auricular fibrillation, which began 30 hours after operation and ceased spontaneously 9½ hours later. There were two anginal attacks during the first fortnight and none subsequently. General massage was begun after five weeks, and she was allowed to wash herself. In six weeks the blood-pressure had returned to 240/112 and was causing symptoms. She is now getting up daily for one to two hours and has kept free of angina. On a balanced diet the blood-pressure has fallen to 210/120. Physical signs of pulmonary collapse have been present at the left base since operation. The electrocardiogram shows no change.

She was allowed gradually to increase her activities, and was eventually discharged to St. Benedict's Hospital on 3rd February, 1937. From the time that she was treated with five grains of quinidine sulphate daily there was only one more brief paroxysm of auricular fibrillation. Her progress at St. Benedict's Hospital was uninterrupted, and she was discharged from there on 18th March. When last seen on 23rd April she was complaining of no symptoms except painful feet. She was able to climb stairs and to go out to church, shopping, etc., and was quite free from anginal pain. She proposed to go home to Hove on 26th April. Her systolic blood-pressure was 220.

In the course of our operations we have made some incidental observations which may be of interest. It is, of course, no new thing for the surgeon to expose and operate on the human heart. Since Rehn demonstrated the possibility of operation for cardiac wounds in 1897

such interventions have become almost commonplace. Before the operation of cardio-omentopexy was devised, although I had exposed and operated on a very large number of animal hearts, my experience of cardiac surgery (as distinct from the surgery of the pericardium) in man had been limited to one or two interventions for stab-wounds, and the dramatic attendant circumstances left me little time for deliberate inspection. In any event the victims of cardiac wounds do not as a rule have diseased hearts.

During our operations we have had occasion to recognize one essential difference between hypertrophy and dilatation of the heart, for in dilatation the coronary vessels are unduly prominent, being forced out from the interventricular sulcus, and we now know that it is necessary to treat such a heart with special care. We have also seen the various types of pericardium—the tense sac over a true hypertrophy which is difficult to incise lest the heart itself be wounded, the slack pericardium over a heart of normal size, and the rather delicate pericardium enclosing a serous effusion. We have also had what is perhaps the unique experience of exposing the heart in a patient suffering from heart block, when we observed cardiac contractions proceed at the rate of 36.

We have also been struck by the greater vascularity of the structures which surround the heart in man as contrasted with the lower animals. The pericardium itself is often highly vascular, and the inferior sternopericardial ligament, insignificant both in the cadaver and in the experimental animal, has been a prominent and richly vascular structure in most of our patients. It is reasonable to suppose that man is under a greater necessity to develop his defences against cardiac ischæmia than are the lower animals, and I have previously pointed out that cardio-omentopexy, as well as bringing a new blood supply into direct relationship with the ischæmic area and the coronary tree, serves to reinforce the normal collaterals in the mediastinum and to give them access to the heart.

### CONCLUSION.

In the present state of knowledge it is impossible to lay down any rigid indications for surgical intervention in cardiac ischæmia. From a consideration of the pathology of the various cardiac disorders it is clear that ischæmia plays an important rôle in many and a dominant rôle in some, and in the Lambeth Clinic we have demonstrated that surgical intervention is practicable and relatively safe, even under rather unfavourable circumstances. Our patients have included a man of 72 and a bed-ridden woman of 65. We are not neglecting the obvious factor of arterial spasm in angina pectoris, but we maintain that spasm is only significant when coronary disease is present. In the laboratory we have paid considerable attention to the nervous factor in coronary obstruction—I am most grateful to Professor Leriche, of Strasbourg, for the personal explanation of his views—and in certain cases we are prepared for operative intervention on the autonomic nervous system.

The selection of patients for operation is in the hands of the medical staff of the Lambeth Cardio-vascular Clinic—Lord Dawson of Penn, Dr. Dan T. Davies, and Dr. H. E. Mansell. We have only operated on a relatively small proportion of the cases sent to us, and in the present stage of the work we have felt it advisable to restrict our interventions to the most severe cases.

In one of his papers Coombs pleaded for the exact correlation of anatomical examination with the clinical findings. At Lambeth we are for the first time able to correlate our clinical findings with what the late Lord Moynihan so well described as the "pathology of the living," and while our researches are only at their beginning, and are in no sense comparable in volume or importance to the vast output which was Coombs's own contribution to cardiology, it may be that in some measure we shall reap where he and other of the great cardiologists have sown.

### REFERENCES.

Allbutt, Sir Clifford, *Diseases of the Arteries*, vol. 2. London: Macmillan, 1915.

Büchner, Franz und Walter v. Lucadou, *Beitr. z. Path. Anat.*, 1934, 93, 165.

Büchner, F., Weber, B., Haager, B., *Koronarinfarkt und Koronarinsuffizienz.* Leipzig: Georg Thieme, 1935.

Chiari, H., *Prag. Med. Wochsch.*, 1897, 22, 61.

Coombs, C. F., *Rheumatic Heart Disease.* Bristol: John Wright, 1924; *Lancet*, 1927, i. 579, 634; Clarke, R. C. *et. al.*, *Quart. J. Med.*, 1927, 21, 51; *Bristol Med.-Chir. J.*, 1927, 144, 249; *Proc. Royal Soc. Med.*, 1928, 21, 727; *Quart. J. Med.*, 1930, 23, 233; *Lancet*, 1930, ii. 227, 281, 333, Aug. 2, 9, 16 (Lumleian Lecture); *Brit. J. Surg.*, 1930, 18, 326; *Bristol Med.-Chir. J.*, 1931, 48, 179; *Quart. J. Med.*, 1932, i. (New Series) 179.

Crainicianu, A., *Virchow's Arch. für Path. Anat.*, 1922, 238, 1.

Fujinami, A., *Virchow's Arch. für Path. Anat.*, 1906, 159, 447.

Grant, P. T., and Viko, L. E., *Heart*, 1929, 15, 103.

Langer, *Bericht d. K. Akad. d. Wissensch.*, Wien, 1880.

Leary, T., and Wearn, J. T., *Amer. Heart J.*, 1930, 5, 412.

Leriche, R., Macewen Memorial Lecture for 1934, Glasgow University, No. 35.

Lewis, Sir Thomas, *Arch. of Int. Med.*, 1932, 49, 713–727.

Mönckeberg, J. G., *Die Erkrankungen des Myocardiums und des spezifischen Muskelsystems, im Handbuch der speziellen Pathologischen Anatomie und Histologie*, F. Henke and O. Lubarsch, Springer, Berlin, 1934.

Moritz, A. R., Hudson, C. L., and Orgren, E. C., *Jour. Expt. Med.*, 1932, 56, 919.

Parkinson, J., and Bedford, D. E., *Heart*, 1928, 14, 194.

von Redwitz, E., *Virchow's Arch. für Path. Anat.*, 1909, 197, 433.

Rehn, L., *Verhand. d. Gesellsch. d. Naturforscher, und Aerzte.*, 1896.

Slater, R., and Kornblum, D., *Jour. Amer. Med. Assoc.*, January, 1934.

Spalteholz, Werner, *Die Arterien der Herzwand.* Leipzig : S. Hirzel, 1924.

Sternberg, M., *Wiener Med. Wchschr.*, 1910, 60, 14.

